# Growth of Single Crystals of (K_1−x_Na_x_)NbO_3_ by the Self-Flux Method and Characterization of Their Phase Transitions

**DOI:** 10.3390/ma17174195

**Published:** 2024-08-24

**Authors:** Doan Thanh Trung, Eugenie Uwiragiye, Tran Thi Lan, John G. Fisher, Jong-Sook Lee, Jungwi Mok, Junseong Lee, Furqan Ul Hassan Naqvi, Jae-Hyeon Ko

**Affiliations:** 1 Department of Materials Science and Engineering, Chonnam National University, 77 Yong-bong ro, Buk-gu, Gwangju 61186, Republic of Korea; doantrung23@gmail.com (D.T.T.); uwiragiye87@gmail.com (E.U.); lantran6393@gmail.com (T.T.L.); 2 Department of Chemistry, Chonnam National University, 77 Yongbong-ro, Buk-gu, Gwangju 61186, Republic of Korea; mokjungwi@naver.com (J.M.); leespy@chonnam.ac.kr (J.L.); 3 School of Semiconductor & Display Technology, Hallym University, 1 Hallymdaehak-gil, Chuncheon 24252, Republic of Korea; furqanhassan05@gmail.com (F.U.H.N.); hwangko@hallym.ac.kr (J.-H.K.)

**Keywords:** (K_0.5_Na_0.5_)NbO_3_, lead-free piezoelectric, single crystal, self-flux crystal growth, Raman scattering, impedance spectroscopy

## Abstract

In this study, single crystals of (K_1−x_Na_x_)NbO_3_ are grown by the self-flux crystal growth method and their phase transitions are studied using a combination of Raman scattering and impedance spectroscopy. X-ray diffraction shows that single crystals have a perovskite structure with monoclinic symmetry. Single crystal X-ray diffraction shows that single crystals have monoclinic symmetry at room temperature with space group P12_1_1. Electron probe microanalysis shows that single crystals are Na-rich and A-site deficient. Temperature-controlled Raman scattering shows that low temperature monoclinic-monoclinic, monoclinic-tetragonal and tetragonal-cubic phase transitions take place at −20 °C, 220 °C and 440 °C. Dielectric property measurements show that single crystals behave as a normal ferroelectric material. Relative or inverse relative permittivity peaks at ~−10 °C, ~230 °C and ~450 °C with hysteresis correspond to the low temperature monoclinic-monoclinic, monoclinic-tetragonal and tetragonal-cubic phase transitions, respectively, consistent with the Raman scattering results. A conduction mechanism with activation energies of about 0.5–0.7 eV was found in the paraelectric phase. Single crystals show polarization-electric field hysteresis loops of a lossy normal ferroelectric. The combination of Raman scattering and impedance spectroscopy is effective in determining the phase transition temperatures of (K_1−x_Na_x_)NbO_3_.

## 1. Introduction

Materials based on (K_0.5_Na_0.5_)NbO_3_ (KNN), a solid solution of ferroelectric KNbO_3_ and anti-ferroelectric NaNbO_3_, are leading contenders to replace lead-based piezoelectric ceramics such as Pb (Zr,Ti)O_3_ and Pb (Mg_1/3_Nb_2/3_)O_3_-PbTiO_3_ [1,2,3,4,5,6]. KNN has moderate piezoelectric properties (d_33_ = 70–90 pC/N, k_p_ = 0.36–0.39, k_t_ = 0.4) [7,8] and much research has focused on improving the piezoelectric properties to make them comparable to Pb (Zr,Ti)O_3_. Towards this end, the phase transitions in the KNbO_3_–NaNbO_3_ system have been studied extensively [1,2,4,9,10,11]. Phase transitions in perovskite ferroelectric materials are of particular interest as the ferroelectric and piezoelectric properties can vary considerably at these transitions [12,13]. The classic example in piezoelectric materials is the enhancement of piezoelectric properties at the morphotropic phase boundary (MPB) between rhombohedral and tetragonal phases in PZT [13,14]. The (K_0.5_Na_0.5_)NbO_3_ composition itself is close to an MPB between two orthorhombic phases [15], or between an orthorhombic and a monoclinic phase [16], leading to an improvement in piezoelectric properties close to this composition [7,17,18]. The majority of research carried out on further improving the properties of KNN has focused on phase boundary engineering, either trying to recreate the rhombohedral/tetragonal MPB in a similar fashion to PZT [19], or by shifting the orthorhombic-tetragonal polymorphic phase transition (PPT) to room temperature [11,20]. Shifting the orthorhombic-tetragonal PPT to room temperature improves the piezoelectric properties, but at a cost of reduced temperature stability of the properties [21,22]. To overcome this problem, research has focused on constructing a rhombohedral-tetragonal phase transition, a rhombohedral-orthorhombic-tetragonal phase transition, or a pseudo-cubic phase at room temperature [23,24,25,26,27].

Alongside research in polycrystalline ceramics, single crystals of KNN and related compositions have also received much attention [20,28,29,30,31,32,33,34,35,36,37,38,39]. Compared to polycrystalline ceramics, single crystals can have improved piezoelectric properties, as they can be oriented in the crystallographic direction with the optimum properties [40,41,42]. Domain engineering can also be used to further improve the properties [14,43]. Single crystals are useful for measuring the intrinsic electrical properties of a material, as contributions from the grain boundaries do not exist [44]. Single crystals are also needed to measure the full set of material constants [34,45]. Single crystals of KNN and related compositions have been grown using several methods, including top-seeded solution growth [20,30,32,33,34,35,36,37], solid-state crystal growth [28], Bridgman growth [38], Czochralski growth [39] and the self-flux method [29,31,36]. The self-flux method is the simplest method of growing single crystals [29,31,36]. A batch, which includes the nominal crystal composition plus an excess of one or more of the components of the crystal (which act as the flux), is heated to above the liquidus temperature to completely melt the batch. After holding the molten batch at constant temperature for several hours to aid homogenization, it is then cooled slowly (1–3 °C/h) through the liquidus temperature into the solid + liquid two-phase region. As the liquid cools, single crystals nucleate from the liquid and grow. After cooling to room temperature, the crystals are removed from the solidified batch and cleaned. This method can only grow small crystals and there is no control over the crystallographic growth direction, but is suitable for growing crystals large enough for property characterization.

In order to successfully carry out phase boundary engineering of KNN, it is necessary to accurately measure the phase transition temperatures. This is often carried out indirectly by measuring the variation of dielectric properties with temperature. Phase transitions are usually marked by peaks in relative permittivity or loss tangent. Raman scattering has also been successfully used to directly study the phase transitions of KNN single crystals, as the Raman spectra undergo significant changes at the phase transitions [46,47,48,49]. In the current work, single crystals of (K_1−x_Na_x_)NbO_3_ are grown using the self-flux method and their phase transitions are studied using a combination of Raman scattering and impedance spectroscopy.

## 2. Materials and Methods

The procedure for growing single crystals of KNN was based on the work of Ray et al. [31]. Powder of composition (K_0.5_Na_0.5_)NbO_3_ was prepared via solid state reaction with K_2_CO_3_ (Daejung, Siheung-si, Republic of Korea >99.5%), Na_2_CO_3_ (Acros Organics, Geel, Belgium, min 99.5%) and Nb_2_O_5_ (Daejung, Siheung-si, Republic of Korea >99.9%) used as starting materials. All starting materials were dried at 250 °C for 5 h to remove adsorbed moisture and then weighed based on the stoichiometric formula. The starting materials were mixed for 24 h by ball mill using high-purity ethanol (99.9%) and ZrO_2_ balls in a polypropylene jar, which was rotated with a speed of 200 rpm. After milling, the slurry was heated on a hotplate/magnetic stirrer to evaporate the ethanol, followed by drying in an oven overnight at 70 °C. The dried slurry was ground in an agate mortar and pestle and passed through a 180 μm sieve to remove agglomerates, followed by calcination at 850 °C for 5 h in air in a covered alumina crucible. The calcined KNN powder was mixed again with excess Na_2_CO_3_ and K_2_CO_3_, at a ratio of 75 weight % (Na_0.5_K_0.5_)NbO_3_: 11.885 weight % K_2_CO_3_: 13.115 weight % Na_2_CO_3_. This corresponds to 25 weight % of (41 mol % K_2_CO_3_: 59 mol % Na_2_CO_3_), which is the eutectic composition in the K_2_CO_3_-Na_2_CO_3_ phase diagram [50]. All materials were dried at 250 °C for 5 h as before. The KNN powder with added flux was ball milled, dried, and ground as above.

**Figure 1:**
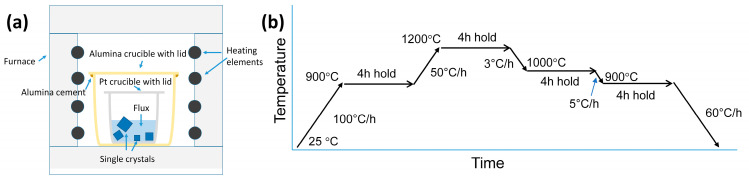
(**a**) Schematic arrangement of the self-flux single crystal growth experiment (reproduced from [51]) (**b**) Heat treatment schedule for single crystal growth.

A Pt crucible was filled with the KNN:K_2_CO_3_:Na_2_CO_3_ powder and closed with a Pt lid. The Pt crucible and lid were placed in an alumina crucible with a lid. The alumina lid was sealed to the alumina crucible with alumina cement (Ceramabond 503, Aremco, Valley Cottage, NY, USA). The cement was fired according to the manufacturer’s instructions. The single crystals were grown in a SiC box furnace. A schematic diagram of the crucible setup is shown in Figure 1a [51]. The furnace thermocouple was positioned several centimeters behind the alumina crucible. According to the manufacturer, the variation in temperature in the furnace chamber’s uniform temperature zone is ±10 °C. The heat treatment schedule for single crystal growth was as follows (Figure 1b): heat from room temperature to 900 °C at a rate of 100 °C/h and hold at 900 °C for 4 h; heat from 900 °C to 1200 °C at a rate of 50 °C/h and hold at 1200 °C for 4 h; cool from 1200 °C to 1000 °C at a rate of 3 °C/h, then hold at 1000 °C for 4 h; cool from 1000 °C to 900 °C at 5 °C/h, then hold at 900 °C for 4 h; cool from 900 °C to 25 °C at 60 °C/h. After cooling, the Pt crucible was placed in boiling water for several days to loosen the solidified flux. The single crystals could then be removed from the flux.

The structure of the grown single crystals was analyzed by XRD (XRD, X’Pert PRO, PANalytical, Almelo, The Netherlands) in Bragg–Brentano geometry using CuKα radiation with a scan range of 20–80° 2θ, a step size of 0.026° and a scan speed of 3°/min. Both bulk and powdered samples were studied. Pattern analysis was carried out using Match! version 3.15 (Crystal Impact, Bonn, Germany) with the Crystallography Open Database. Single crystal XRD was carried out using a Bruker APEX-II CCD-based diffractometer (Bruker AXS GmbH, Karlsruhe, Germany) with graphite-monochromated MoKα radiation (λ = 0.71073 Å). The hemisphere of the reflection data was collected as ω scan frames at 0.5°/frame and an exposure time of 5 s/frame. The cell parameters were determined and refined using the APEX2 program [52]. The data were corrected for Lorentz and polarization effects and an empirical absorption correction was applied using the SADABS program [53]. The compound structures were solved by direct methods and refined by full matrix least-squares using the SHELXTL program package (version number 2014-3) [54] and Olex2 (version 1.5) [55] with anisotropic thermal parameters for all non-hydrogen atoms. Electron probe microanalysis was carried out on the single crystal to determine its composition as described below. Due to the small size of the single crystal, it was not polished before analysis.

Electron probe microanalysis (EPMA, JEOL JXA-8530F PLUS, Tokyo, Japan) was carried out on selected single crystals to determine their chemical composition. Samples were polished to a 1 μm finish using diamond paste. Wavelength-dispersive spectroscopy analysis was carried out on carbon-coated samples using an accelerating voltage of 15 kV. NaAlSi_2_O_6_ and KNbO_3_ were used as standards. The surfaces of as-grown crystals were examined with a scanning electron microscope (SEM, S-4700, Hitachi, Tokyo, Japan) equipped with an Energy Dispersive X-ray Spectrometer (EDS, Horiba EMAX Energy EX-200, Kyoto, Japan) using standardless analysis. Samples for SEM were Pt-coated.

The phase transitions of a single crystal were studied using Raman scattering. A single crystal was polished to a 1 µm finish with diamond suspension, annealed for 1 h at 600 °C to remove polishing strains and cooled at a rate of 1 °C/min. Raman spectra were measured upon heating from −198 °C to 600 °C using a heating stage (THMS600, Linkam, Tadworth, UK) in the wavenumber range from 10 to 1000 cm^−1^. The spectral resolution of the system was 1~2 cm^−1^. The diameter of the laser beam on the single crystal surface was 1~2 µm and the laser wavelength was 532 nm. All spectra are corrected by the Bose–Einstein factor [56].

For the impedance spectroscopy measurements, a single crystal sample was parallel polished on both major faces with SiC paper up to grade #4000 and Ag paste electrodes (16032 PELCO, Ted Pella, Redding, CA, USA) were applied. The thickness of the sample was 0.1388 cm and the electrode area was 0.2112 cm^2^. The sample was loaded in a heating stage (THMS600, Linkam, Tadworth, UK) and connected to Pt wires using Pt paste. Dielectric constant, loss tangent and conductivity were measured upon heating and cooling in the temperature range between −190 °C and 590 °C with heating and cooling rates of 1 °C/min using an impedance analyzer (HP4284A, Hewlett-Packard, Kobe, Japan). For ferroelectric property measurements, a single crystal sample was parallel polished on both major faces with SiC paper up to grade #4000. Ag electrodes (DS-PF-7472, Daejoo, Siheung-si, Republic of Korea) were applied and fired at 600 °C for 1 h. The sample had a thickness of 0.1073 cm and an area of 0.0502 cm^2^. Polarization vs. electric field hysteresis loops were measured in silicon oil at room temperature at a frequency of 20 Hz using a Sawyer Tower circuit (Multiferroic combined with 4 kV High Voltage Interface, Radiant Technologies Inc., Albuquerque, NM, USA).

## 3. Results and Discussion

**Figure 2:**
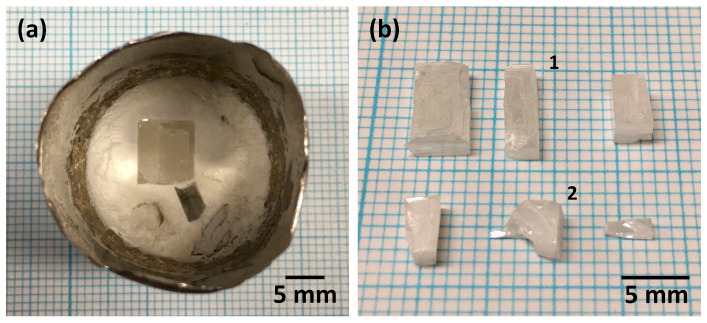
(K_1−x_Na_x_)NbO_3_ single crystals (**a**) in the Pt crucible and (**b**) after removal from the crucible and cleaning.

**Figure 3:**
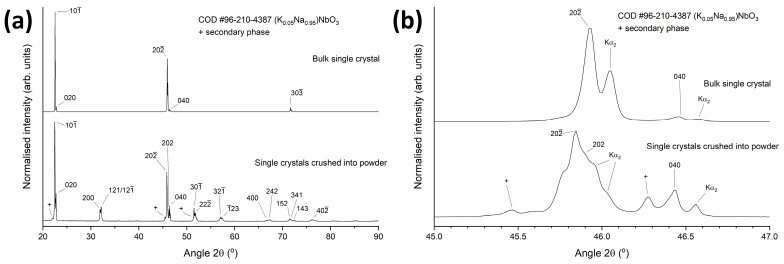
(**a**) XRD patterns of a bulk (K_1−x_Na_x_)NbO_3_ single crystal and (K_1−x_Na_x_)NbO_3_ single crystals crushed into powder; (**b**) the patterns in the 2θ range 45–47°.

Figure 2 shows KNN single crystals in the Pt crucible and after removal from the Pt crucible. Single crystals up to 8 mm × 5 mm in size could be grown. The single crystals are opaque, possibly due to the scattering of light by ferroelectric domains [57]. Single crystals numbered 1 and 2 were examined by SEM. Figure 3 illustrates XRD patterns of a bulk KNN single crystal and crushed KNN single crystals, respectively. The patterns in Figure 3a can be indexed with Crystallography Open Database pattern #96-210-4387 for (K_0.05_Na_0.95_)NbO_3_ (monoclinic, space group P1m1). The $10\bar{1}$, $20\bar{2}$ and $30\bar{3}$ reflections of the bulk KNN single crystal are very strong, with weaker 020 and 040 reflections also present. Other reflections are absent. For an X-ray diffractometer in Bragg–Brentano geometry, only crystallographic planes parallel to the sample surface can diffract X-rays [58]. Intense reflections appear only from parallel crystallographic planes, indicating that the sample is a single crystal. The appearance of the 020 and 040 reflections indicate the existence of non-180° ferroelectric/ferroelastic domains [59,60,61]. The XRD pattern of the crushed KNN single crystals is similar to that of a polycrystalline material, as expected. Small amounts of a secondary phase are present. This is probably flux that was not removed from the crystal surfaces. Figure 3b shows the peaks in the region 45–47° 2θ. It can be seen that the 2θ values of the reflections of the bulk single crystal are slightly different than those of the crystals that were crushed into powder. To prepare the powder sample, several smaller crystals were crushed into powder. Different single crystals may have different chemical compositions and hence different lattice parameters, leading to the shift in peak positions. This may also explain the shoulder on the low angle side of the $20\bar{2}$ reflection.

**Figure 4:**
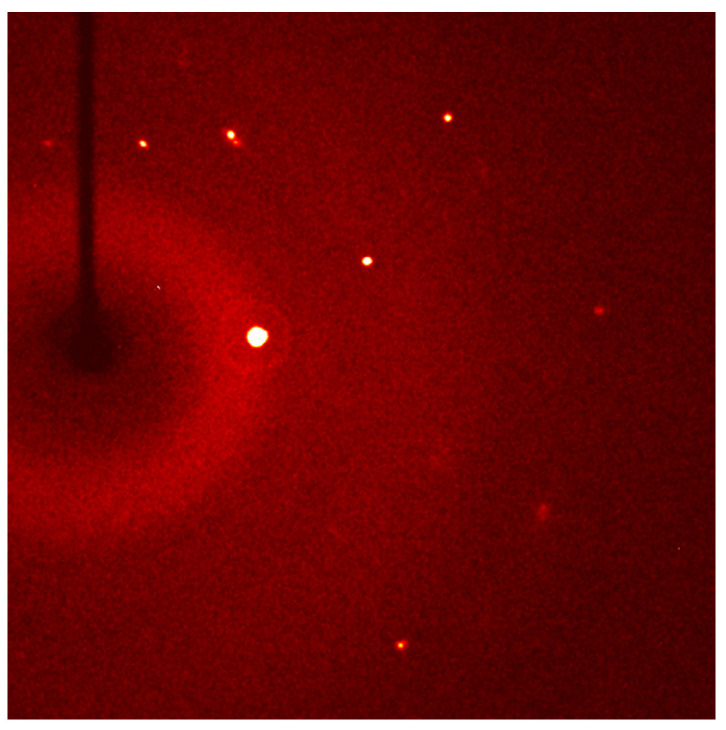
Single crystal XRD pattern of a bulk (K_1−x_Na_x_)NbO_3_ single crystal.

A diffraction pattern of a KNN single crystal taken using single crystal XRD is shown in Figure 4. The diffraction pattern shows that the sample really is a single crystal. The unit cell parameters are given in Table 1. The crystal system is monoclinic, as also found by powder XRD. Note that the space group found by single crystal XRD in this work is P12_1_1, which differs from the space group of the Crystallography Open Database pattern used above. The crystal data and structure refinement of the single crystal are given in Table S1. The chemical composition of this crystal as analyzed by EPMA is given in Table S2. The results are shown as the mean mol % and standard deviation of each constituent oxide. Ten measurements were made on this crystal. The formula unit of the single crystal can be written as (K_0.25±0.01_Na_0.62±0.02_)NbO_3_. The single crystal is Na-rich compared to the nominal composition. It is also alkali-deficient compared to the nominal composition. Due to its small size (1~2 mm), this sample was not polished before analysis. This may explain the large values of standard deviation. For the analysis of the single crystal XRD data, the above formula unit was initially used, but it was found that a formula unit of (K_0.30_Na_0.62_)NbO_3_ gave lower R-values. Possibly the surface and bulk compositions of the single crystal differ.

The chemical composition of two single crystals was analyzed by EPMA (Table 2). The results are shown as the mean mol % and standard deviation of each constituent oxide. Ten measurements were made on single crystal A and 20 measurements were made on single crystal B. The two crystals have different compositions. The formula units of the two single crystals can be written as (K_0.21±0.00_Na_0.67±0.01_)NbO_3_ and (K_0.15±0.00_Na_0.80±0.01_)NbO_3_, respectively. Both crystals are Na-rich compared to the nominal composition and are alkali-deficient in total. The partition coefficients of Na and K were calculated to be >1 and <1, respectively, by Koruza et al., meaning that Na is expected to be preferentially incorporated into the crystal [36]. Single crystals of (K_1−x_Na_x_)NbO_3_ grown by flux growth, top-seeded solution growth and Czochralski growth were also found to be Na-rich compared to their nominal compositions [39,57,62]. Ahtee and Glazer found that the composition of KNN single crystals grown by the flux method (using NaF as a flux) could vary within a batch over a range of 30 mol % [62]. The amount of K in the crystal can be increased by raising the K content of the flux or the starting KNN powder [36,62]. The reason for the deficiency in total alkali content is not clear. It may be due to the higher volatility of K compared to Na [63]. The difficulty of accurately measuring alkali elements by EPMA may also be a reason [64]. However, the same EPMA equipment was previously successfully used to measure single crystals of nominal (K_0.5_Na_0.5_)NbO_3_ composition [65]. The compositions of the two single crystals differ from each other, as well as from the single crystal used for single crystal XRD (Table S2). This may be caused by variations in temperature or composition in different regions of the flux. Compositional variations could be due to insufficient homogenization of the flux or by alkali evaporation from the surface of the flux. It is worth noting that for compositions with >55 mol % NaNbO_3_ the low temperature phase of KNN is monoclinic rather than rhombohedral as is the case for (K_0.5_Na_0.5_)NbO_3_ [15].

**Figure 5:**
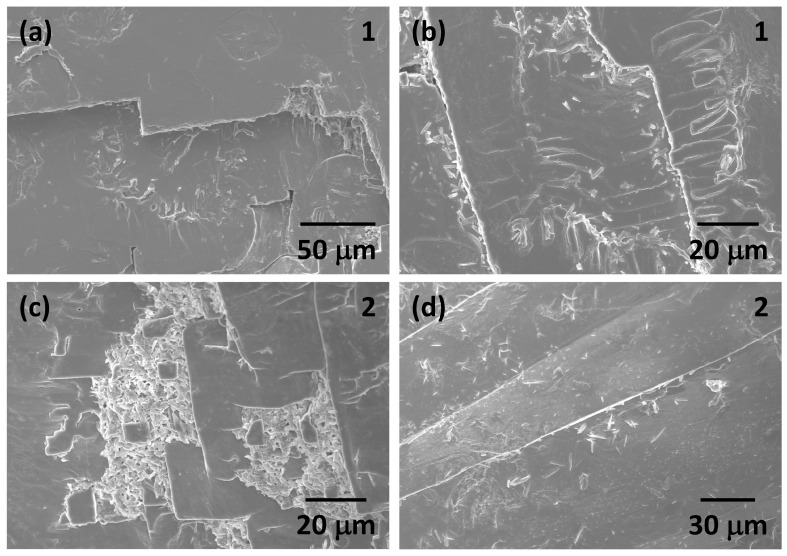
SEM micrographs of as-grown surfaces of (K_1−x_Na_x_)NbO_3_ single crystals: (**a**,**b**) single crystal 1; (**c**,**d**) single crystal 2 in Figure 2.

SEM micrographs of the growth surfaces of two single crystals (numbered 1 and 2 in Figure 2) are shown in Figure 5. Multiple growth steps and ledges can be seen on the surface of the single crystals, indicating that they have grown by polynucleation and growth. EDS analysis shows that the single crystals contain more Na than K. Secondary phases are present on the surface of the single crystals. EDS analysis shows that these phases contain Na, K, Nb and O. The acicular secondary phase in Figure 5a,b has a similar composition to the single crystal. The secondary phase in Figure 5c contains more K than Na and may be solidified flux. The needle-like phase in Figure 5d could not be measured by EDS as the sample surface was at too steep an angle. 

**Figure 6:**
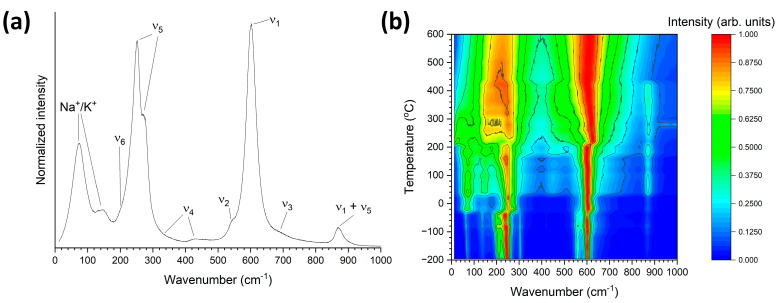
(**a**) Raman spectrum of a single crystal of (K_1−x_Na_x_)NbO_3_ taken at 20 °C; (**b**) Normalized intensity contour plot of Raman spectra of a (K_1−x_Na_x_)NbO_3_ single crystal taken at different temperatures.

Figure 6a shows a Raman spectrum of a (K_1−x_Na_x_)NbO_3_ single crystal taken at 20 °C. The spectrum is characteristic of the orthorhombic phase of (Na_0.5_K_0.5_)NbO_3_ [66,67]. In the discussion of the XRD results above, the unit cell was indexed as monoclinic. In the literature, the room temperature unit cell of KNN is usually described as orthorhombic, although it is actually monoclinic. Based on the choice of axes, the KNN unit cell can be identified as either monoclinic or orthorhombic [36,59,68]. The peaks of the Raman spectrum can be assigned based on their similarity to the patterns of KNbO_3_ and NaNbO_3_ [66,69,70,71]. The major peaks in the spectrum can be assigned to the internal modes of the NbO_6_ octahedra with O_h_ point group [66,72]. There are three Raman active modes (ν_1_ (A_1g_), ν_2_ (E_g_) and ν_5_ (F_2g_)), which are expected to show strong scattering, while three Raman inactive modes (ν_3_ (F_1u_), ν_4_ (F_1u_) and ν_6_ (F_2u_)) may be detected as weak scattering [67,73]. The peaks in the range 0–200 cm^−1^ are assigned to the translational modes of K^+^/Na^+^ cations. The peak at ~250 cm^−1^ can be assigned to the NbO_6_ ν_5_ bending mode. The shoulder on the left of this peak can be assigned to the ν_6_ mode. The shoulder on the right of the peak at ~250 cm^−1^ and the small peak at ~430 cm^−1^ are assigned to the ν_4_ bending mode. The peak at ~600 cm^−1^ and the shoulder at ~550 cm^−1^ are assigned to the NbO_6_ ν_1_ and ν_2_ stretching modes, respectively. The shoulder to the right of the ν_1_ peak at ~700 cm^−1^ is assigned to the ν_3_ stretching mode. The peak at ~870 cm^−1^ is assigned to a combination tone of the ν_1_ and ν_5_ modes. Figure 6b shows an intensity contour plot of the normalized intensity of the spectra (each spectrum was normalized to its maximum intensity). It was found that normalizing the spectra showed the changes in the spectra with temperature more clearly than using unnormalized spectra. Changes in the positions, widths and relative intensities of the strongest peaks (ν_1_ and ν_5_ modes) can be seen at −20 °C, 220 °C and 440 °C. These two modes, along with the ν_2_ mode, are sensitive to phase transitions, as they are internal modes [49].

**Figure 7:**
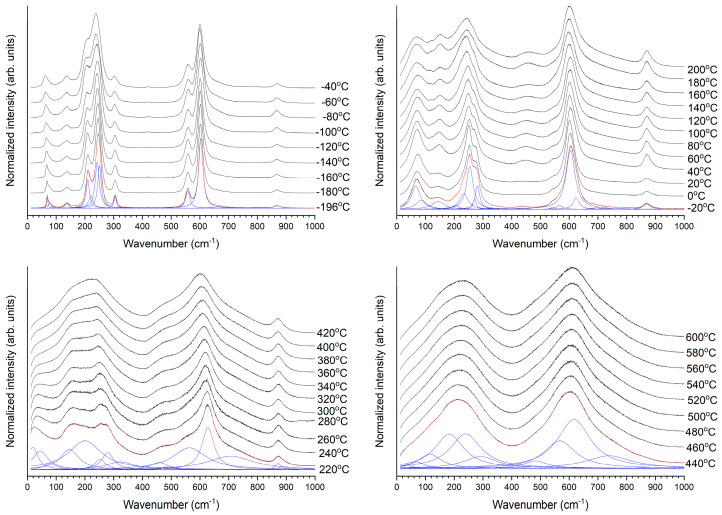
Normalized Raman spectra of a (K_1−x_Na_x_)NbO_3_ single crystal taken at different temperatures. Representative spectra are shown with fitted Lorentzian peaks in blue. The red curves are the sum of the fitted peaks.

Figure 7 shows the normalized Raman spectra taken at different temperatures. The spectra look similar to those of KNbO_3_ and (K_0.5_Na_0.5_)NbO_3_ [67,74] rather than those of NaNbO_3_ [75,76]. All of the spectra were fitted with Lorentzian peaks using Origin Pro 2023 (OriginLab Corporation, Northampton, MA, USA). Fitted peaks for representative spectra are shown in blue. The red curves are the sum of the fitted peaks. Clear changes in the shape of the spectra, the number, position and shape of the fitted peaks can be seen at −20 °C, 220 °C and 440 °C. In general, the modes become broader and merge together as temperature increases. The relative intensities and FWHM of the Na^+^/K^+^ modes between 60–100 cm^−1^ steadily increase with temperature between −196 °C and −40 °C, with a further abrupt increase at −20 °C. The modes shift to lower wavenumbers at 220 °C and then gradually decrease in relative intensity. The Na^+^/K^+^ mode at 138 cm^−1^ also increases in relative intensity and FWHM with increasing temperature. A low-wavenumber shoulder (118 cm^−1^) appears on this peak at 40 °C, increases in intensity to form a separate peak, then disappears at 220 °C. The Na^+^/K^+^ mode at 138 cm^−1^ abruptly increases in relative intensity and FWHM at 220 °C. Above 400 °C, its relative intensity decreases. The relative intensity of the ν_6_ modes between 180–240 cm^−1^ steadily decrease with temperature, undergo an abrupt decrease at −20 °C and almost completely merge with the ν_5_ peak before suddenly increasing in FWHM and relative intensity at 200 °C. As temperature increases further, the Na^+^/K^+^ modes merge with the ν_6_ and ν_5_ peak. A shoulder appears on the right of the ν_5_ peak at −20 °C, gradually merging with the peak as temperature increases.

The ν_4_ mode at 305 cm^−1^ gradually weakens in intensity as temperature increases, eventually disappearing at 440 °C. Conversely the ν_4_ mode at 422 cm^−1^ initially has low relative intensity, suddenly increases in FWHM and intensity at 40 °C and then gradually merges with the ν_2_ mode and reduces in intensity above 200 °C before disappearing at 440 °C. The ν_2_ peak is initially separate from the intense ν_1_ peak, but decreases in intensity and merges with the ν_1_ peak. At −20 °C, its intensity abruptly decreases and it becomes a shoulder of the ν_1_ peak. At 220 °C, it shifts to lower wavenumbers and increases in intensity. The ν_1_ peak broadens with increasing temperature. A second ν_1_ peak appears at −20 °C and steadily increases in intensity, becoming more intense than the first ν_1_ peak at 180 °C. At 220 °C, the two ν_1_ peaks shift in position and the lower wavenumber peak increases in FWHM. The higher wavenumber ν_1_ peak becomes broader with increasing temperature. The ν_3_ mode slowly increases in intensity with temperature, undergoing a sudden increase in intensity and FWHM at 220 °C. It begins to merge with the ν_1_ peak at 440 °C. The ν_1_ + ν_5_ peak increases in intensity with temperature, becoming asymmetric in shape. A second ν_1_ + ν_5_ peak appears at 0 °C. The ν_1_ + ν_5_ peaks appear to abruptly disappear at 440 °C.

**Figure 8:**
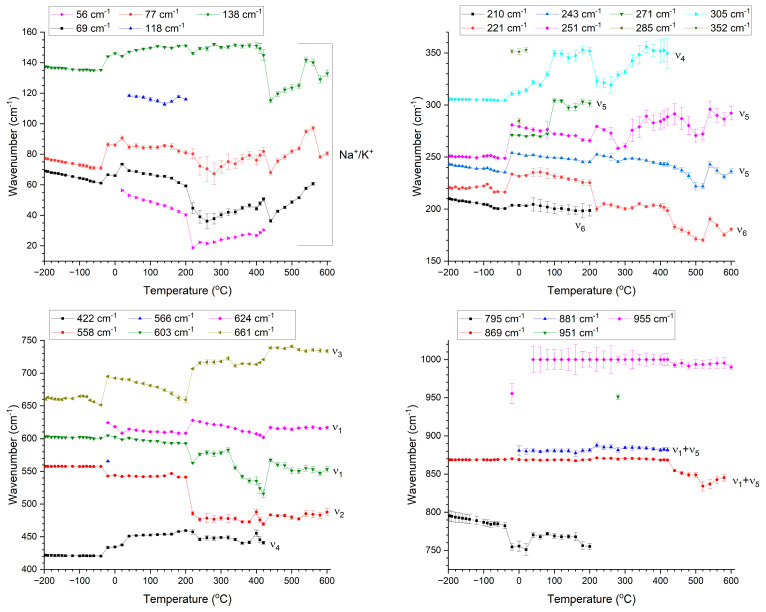
Position of the Raman modes vs. temperature. Each mode is labelled with the value of its wavenumber at −196 °C or the temperature at which it first appeared.

Figure 8 shows the peak positions of the Raman modes vs. temperature. Each mode is labelled with the value of its wavenumber at −196 °C or the temperature at which it first appeared. The major modes have also been labelled according to the discussion in the previous paragraphs. The FWHM and peak area of the modes vs. temperature are shown in Figures S1 and S2. The error bars are the standard error as calculated by Origin Pro. Sudden shifts in mode position are visible at −20 °C, 220 °C and 440 °C. In addition, some modes appear and others disappear at or close to these temperatures. Some of the modes also show a change in behavior between 260 and 320 °C. Discontinuous changes in the FWHM and peak area of the modes also occur at these temperatures (Figures S1 and S2). The mode at 795 cm^−1^ is initially very weak and part of the background. It increases in intensity before disappearing at 220 °C. The ν_1_ + ν_5_ peak doesn’t entirely disappear at 440 °C but merges into the background. The second ν_1_ + ν_5_ peak that appeared at 0 °C disappears at 440 °C. The mode at ~1000 cm^−1^ is added to fill in the background.

The number and relative intensity of the Raman modes is determined by the point group of the crystal [77,78], which makes Raman scattering useful for studying phase transitions. Group theory indicates 7 Raman active modes (3A_1_ + 4E) for the rhombohedral phase, 12 Raman active modes (4A_1_ + A_2_ + 4B_1_ + 3B_2_) for the orthorhombic phase, 8 Raman active modes (3A_1_ + B_1_ + 4E) for the tetragonal phase and 4 Raman inactive modes (3F_1u_ + F_2u_) for the cubic phase [66,71,74]. From Figure 7, it can be seen that the number of modes for each phase is greater than that predicted by group theory analysis, a common occurrence for ferroelectric perovskites [74]. Distortion of the NbO_6_ octahedra and a deviation from O_h_ symmetry can cause broadening or splitting of the modes [72,79]. If a degenerate mode is polar, its degeneracy can by lifted, causing extra Raman peaks to appear [72,77]. The changes in mode position, FWHM and area at −20 °C, 220 °C and 440 °C, plus the appearance and disappearance of modes, are consistent with rhombohedral-orthorhombic, orthorhombic-tetragonal and tetragonal-cubic phase transitions taking place. It was mentioned earlier that the low temperature phase for these single crystals is monoclinic [15]. The low temperature Raman spectra are similar to those of the rhombohedral phase of KNbO_3_ and (K_0.5_Na_0.5_)NbO_3_ [71,74]. The single crystals may have different structures at different length scales, as is often the case for ferroelectric materials [80,81,82,83,84,85,86]. 

**Figure 9:**
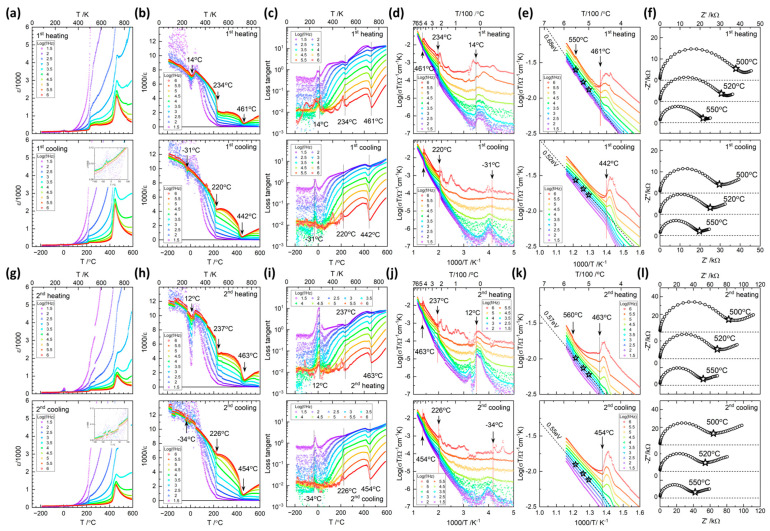
(**a**) ε, (**b**) ε^−1^, (**c**) tan δ, (**d**), (**e**) σT vs. temperature of a (K_1−x_Na_x_)NbO_3_ single crystal on 1st heating and cooling: (**g**) ε, (**h**) ε^−1^, (**i**) tan δ, (**j**), (**k**) σT on 2nd heating and cooling. (**f**,**l**) are the respective impedance spectra at selected temperatures as indicated by stars in (**e**,**k**). Phase transition temperatures from the dielectric peaks are indicated for all plots.

The relative permittivity, dielectric loss tangent, and the ac conductivity for a sample measured over two cycles between −190 °C and 590 °C are presented in Figure 9. The numbers in the legends represent logarithmic frequencies, i.e., 6 for 10^6^ Hz, 5.5 for 10^5.5^ or 316,227 Hz, etc. The results are similar to those of KNN single crystals and ceramics found by other researchers [57,87,88,89]. The low temperature monoclinic-monoclinic phase transition is not clearly visible in the relative permittivity plots (see insets in the cooling plots of Figure 9a,g). The peaks that appear at ~0 °C in the permittivity plots on heating may be artefacts caused by melting of water vapor in the sample chamber that had frozen during cooling [90]. Figure 9b,h, inverse permittivity plots, show the small dielectric peaks at low temperature more clearly, as indicated. The two cycles show similar transition temperatures with hysteresis at −10 (±25) °C, 230 (±10) °C and 450 (±10) °C, which is consistent with those in the Raman spectra. The temperatures are indicated in the loss tangent and also AC conductivity plots. As tan δ = ε″/ε′, similar cusps appear as ε′^−1^ at 450 (±10) °C. As σ ∝ ε″, corresponding behavior is shown in the AC conductivity plots modified by the T term. The low temperature monoclinic-monoclinic transitions around −10 °C are shown to display strong effects in the low-frequency ε″ or equivalently tan δ and σ, suggesting conduction loss. 

As shown in the spectra at 500, 520 and 550 °C (Figure 9f,l), the AC conductivity curve at 1kHz corresponds to the bulk conductivity, with an activation energy of ~0.6 (±0.08) eV, which is smaller than previously reported. Blocking effects in the low frequency with Ag electrodes suggests the contribution of ionic conduction by the movement of potassium and oxygen vacancies [87,91]. The conduction mechanism appears to persist below the transition at 450 (±10) °C. Large variation in low frequency tan δ and σ near the low temperature monoclinic-monoclinic transition should be related with condensation and freezing of humidity in the ambient air, leading to protonic surface conduction [46,47,90]. Whether the two phenomena are concomitant or accidental needs to be clarified. 

On heating, a transition around 555 °C is indicated in the AC conductivity plots, which could be the Burns temperature, representing the diminishing small ferroelectric regions above T_C_. Raman spectra show similar effects. The 2nd cycle, Figure 9g–l, reduces the dielectric constant values as shown in the ε′ and ε′^−1^ plots. The peak value at 450 (±10) °C is reduced to from 2000 to 1000 and conductivity above 450 (±10) °C is also reduced by half. 

**Figure 10:**
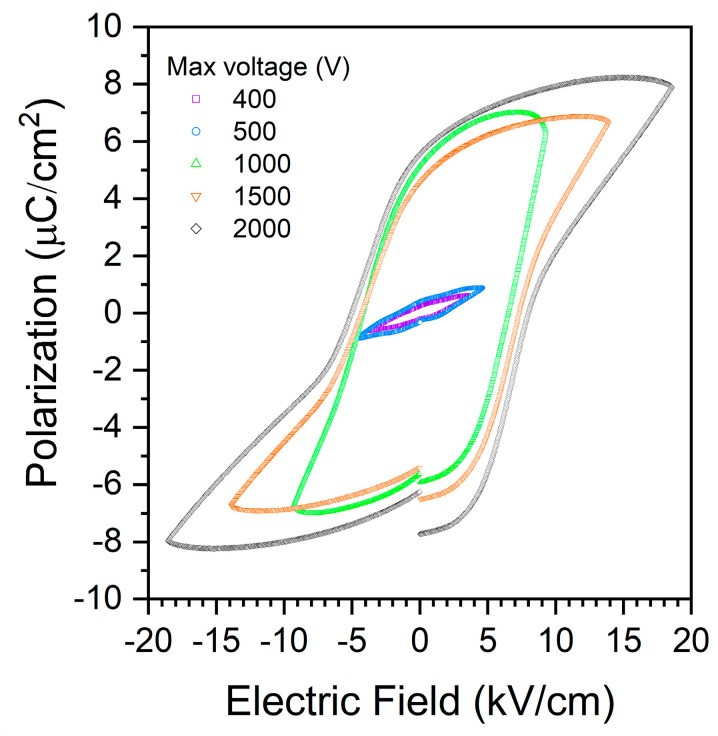
Polarization vs. electric field hysteresis loops of a (K_1−x_Na_x_)NbO_3_ single crystal.

A polarization vs. electric field (PE) hysteresis loop of a sample is shown in Figure 10. The PE loops begin to saturate under an electric field of 10 kV/cm. The samples show PE loops typical of a lossy normal ferroelectric material [92]. The sample has very low polarization even under an electric field of 20 kV/cm, indicating that the electric field is not sufficient to cause significant switching of the domains. The gaps between the end and start of each hysteresis loop also indicate that the sample is semiconducting [92].

## 4. Conclusions

(K_1−x_Na_x_)NbO_3_ single crystals were grown by the self-flux method. The single crystals have monoclinic symmetry at room temperature with space group P12_1_1 and are Na-rich and A-site deficient. A combination of impedance spectroscopy and temperature-controlled Raman scattering was used to determine the low temperature monoclinic-monoclinic, monoclinic-tetragonal and tetragonal-cubic phase transitions. Peaks in relative permittivity at ~240 °C and ~465 °C correspond to the monoclinic-tetragonal and tetragonal-cubic phase transitions, respectively, while anomalous changes in the Raman spectra show that the phase transitions take place at 220 °C and 440 °C. The low temperature monoclinic-monoclinic phase transition could not be easily detected on the plots of relative permittivity vs. temperature, but could be observed by Raman scattering and be detected on the cooling plots of inverse relative permittivity, loss tangent and conductivity vs. temperature. The sample has polarization vs. electric field hysteresis loops typical of a lossy normal ferroelectric material, but with low polarization. The combination of impedance spectroscopy and temperature-controlled Raman scattering is very useful for determining the phase transition temperatures, especially for phase transitions which are difficult to detect by one method alone.

## Figures and Tables

**Table 1:** Unit cell parameters of a (K_1−x_Na_x_)NbO_3_ single crystal.

Space Group Crystal System	Monoclinic
Space group name	P12_1_1
Unit cell length a	3.9212(4) Å
Unit cell length b	5.5795(5) Å
Unit cell length c	5.5712(5) Å
Unit cell angle α	90°
Unit cell angle β	90.004(5)°
Unit cell angle γ	90°
Unit cell volume	121.89(2) Å^3^
Formula units (Z)	2

**Table 2:** Chemical composition of two (K_1−x_Na_x_)NbO_3_ single crystals as measured by EPMA.

Oxide	Single Crystal A (mol %)	Single Crystal B (mol %)	Nominal Composition (mol %)
K_2_O	11.15 ± 0.08	7.49 ± 0.13	25
Na_2_O	35.64 ± 0.38	41.10 ± 0.32	25
Nb_2_O_5_	53.21 ± 0.40	51.41 ± 0.30	50

## Data Availability

The original contributions presented in the study are included in the article/Supplementary Materials, further inquiries can be directed to the corresponding author/s.

## Supplementary Materials

The following supporting information can be downloaded at: https://www.mdpi.com/article/10.3390/ma17174195/s1, Figure S1: FWHM of the Raman modes vs. temperature. Each mode is labelled with the value of its wavenumber at −196 °C or the temperature at which it first appeared; Figure S2: Peak area of the Raman modes vs. temperature. Each mode is labelled with the value of its wavenumber at −196 °C or the temperature at which it first appeared; Table S1: Crystallographic data and parameters for a (K_1−x_Na_x_)NbO_3_ single crystal; Table S2: Chemical composition of the (K_1−x_Na_x_)NbO_3_ single crystal used for single crystal XRD.
