# Supplementary material for: Growth of Single Crystals of (K1−xNax)NbO3 by the Self-Flux Method and Characterization of Their Phase Transitions
Source: Materials (Basel). 2024 Aug 24;17(17):4195. doi: 10.3390/ma17174195 (PMC11396741; doi:10.3390/ma17174195)
Supplement: Supplementary file 1 [file materials-17-04195-s001.zip › materials-3106376-supplementary.pdf]

Growth of Single Crystals of $(K_{1-x}Na_x)NbO_3$ by the Self-Flux Method and Characterization of Their Phase Transitions

Doan Thanh Trung [1], Eugenie Uwiragiye [1,†], Tran Thi Lan [1], John G. Fisher [1,*], Jong-Sook Lee [1,*], Jungwi Mok [2], Junseong Lee [2], Furqan Ul Hassan Naqvi [3] and Jae-Hyeon Ko [3]

[1] Department of Materials Science and Engineering, Chonnam National University, 77 Yong-bong ro, Buk-gu, Gwangju 61186, Republic of Korea; doantrung23@gmail.com (D.T.T.); uwiragiye87@gmail.com (E.U.); lantran6393@gmail.com (T.T.L.)

[2] Department of Chemistry, Chonnam National University, 77 Yongbong-ro, Buk-gu, Gwangju 61186, Republic of Korea; mokjungwi@naver.com (J.M.); leespy@chonnam.ac.kr (J.L.)

[3] School of Semiconductor & Display Technology, Hallym University, 1 Hallymdaehak-gil, Chuncheon 24252, Republic of Korea; furqanhassan05@gmail.com (F.U.H.N.); hwangko@hallym.ac.kr (J.-H.K.)

* Correspondence: johnfisher@jnu.ac.kr (J.G.F.); jongsook@jnu.ac.kr (J.-S.L.); Tel.: +82-62-530-1702 (J.G.F.); +82-62-530-1701 (J.-S.L.)

† Current address: Department of Mechanical and Energy Engineering, University of Rwanda-College of Science and Technology, Kigali P.O. Box 3900, Rwanda.

Supplementary Materials

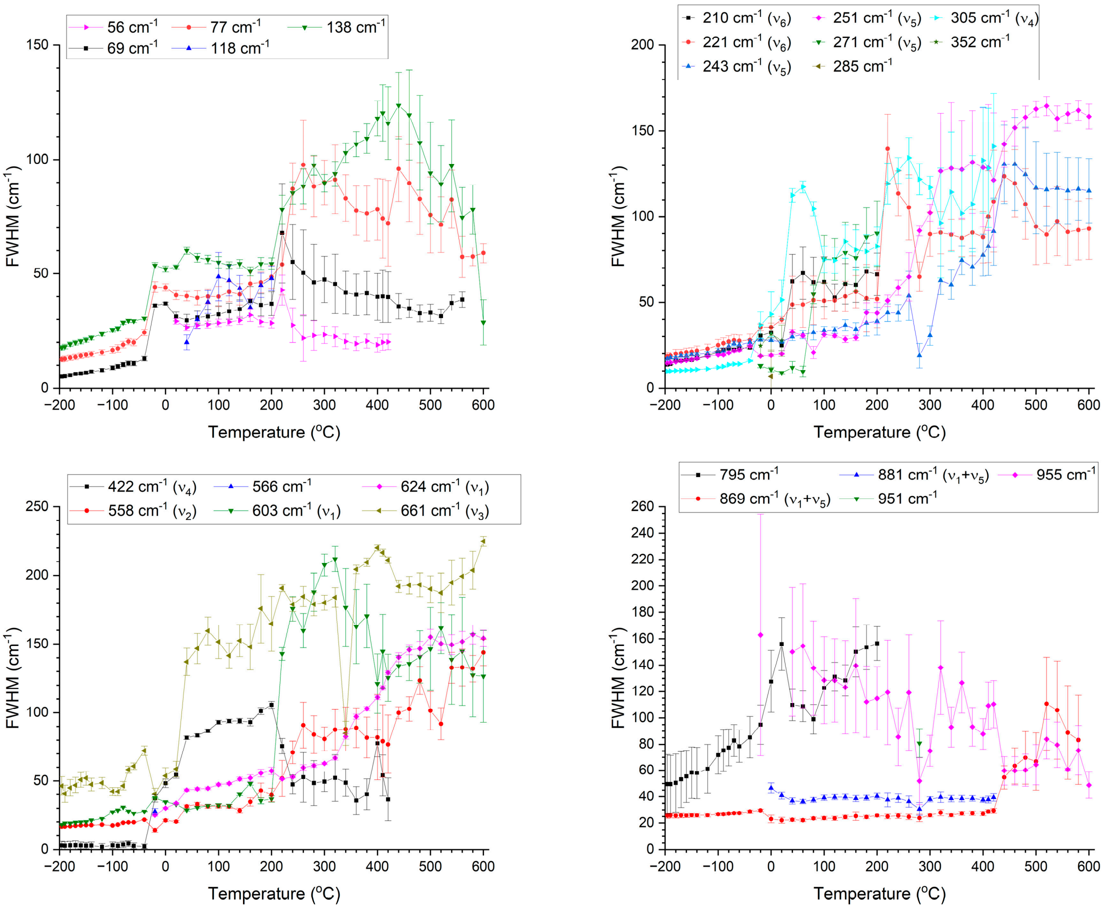

Figure S1: FWHM of the Raman modes vs. temperature. Each mode is labelled with the value of its wavenumber at -196°C or the temperature at which it first appeared.

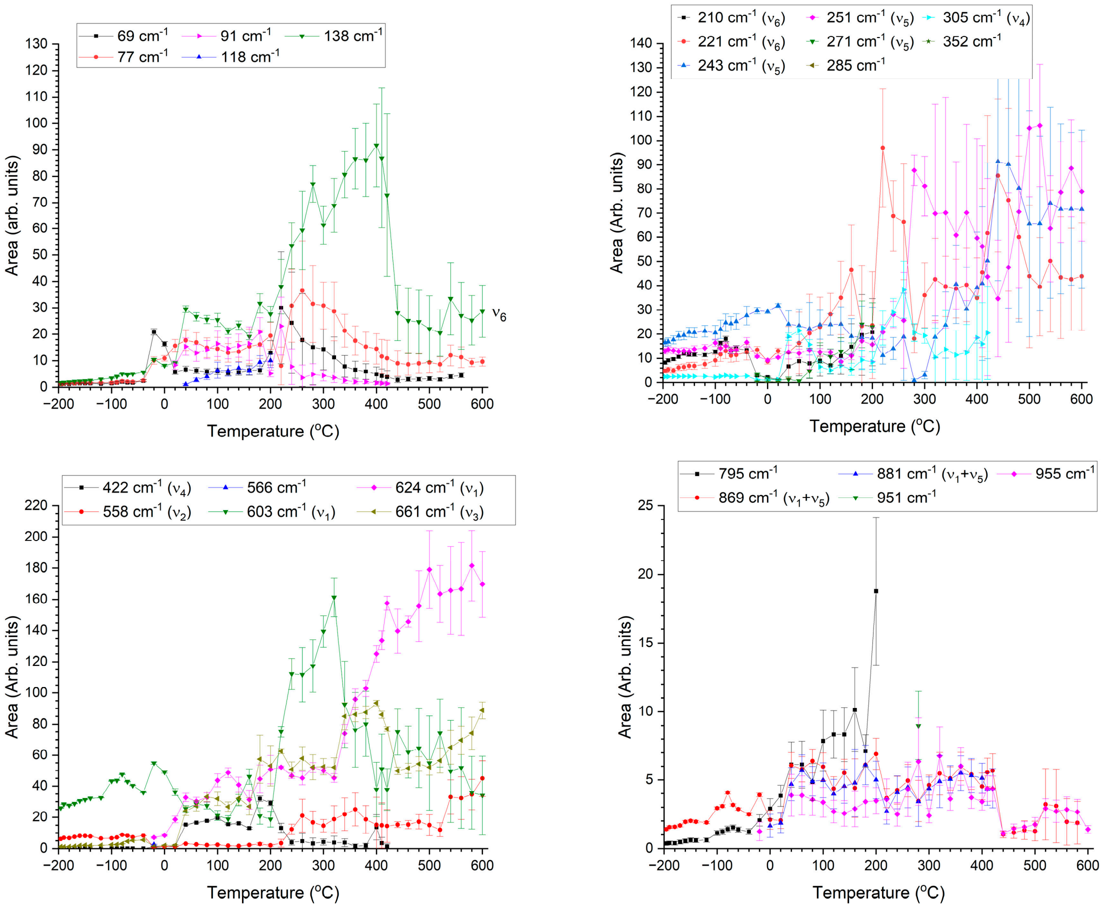

Figure S2: Peak area of the Raman modes vs. temperature. Each mode is labelled with the value of its wavenumber at -196°C or the temperature at which it first appeared.

Table S1: Crystallographic data and parameters for a $(K_{1-x}Na_x)NbO_3$ single crystal.

Formula unit	$K_{0.30}Na_{0.62}NbO_3$
Formula weight	166.90
Temperature/K	298
Crystal system	monoclinic
Space group	$P12_11$
a/Å	3.9212(4)
b/Å	5.5795(5)
c/Å	5.5712(5)
α/°	90
β/°	90.004(5)
γ/°	90
Volume/$Å^3$	121.89(2)
Z	2
ρ_{calc}g/cm^3	4.547
μ/mm^{-1}	5.274
F(000)	155.0
Crystal size/mm^3	0.05 × 0.05 × 0.05
Radiation	MoKα (λ = 0.71073)
2Θ range for data collection/°	7.314 to 50.842
Index ranges	$-4 \le h \le 4$, $-6 \le k \le 6$, $-6 \le l \le 6$
Reflections collected	2443
Independent reflections	462 [R_{int} = 0.0354, R_{sigma} = 0.0233]
Data/restraints/parameters	462/19/52
Goodness-of-fit on F^2	1.154
Final R indexes [I>=2σ (I)]	[a] R_1 = 0.0438, [b] wR_2 = 0.1024
Final R indexes [all data]	[a] R_1 = 0.0451, [b] wR_2 = 0.1036
Largest diff. peak/hole / e $Å^{-3}$	1.69/-2.45
Flack parameter	0.48(6)

[a] $R_1 = \sum||Fo| - |Fc||/\sum|Fo|$. [b] $wR_2 = \{[\sum w(Fo^2 - Fc^2)^2]/[\sum w(Fo^2)^2]\}^{1/2}$.

Table S2: Chemical composition of the $(K_{1-x}Na_x)NbO_3$ single crystal used for single crystal XRD.

Oxide	Composition (mol %)	Nominal composition (mol %)
K_2O	13.50 ± 1.02	25
Na_2O	32.68 ± 2.17	25
Nb_2O_5	53.81 ± 1.23	50
